# The Impact of Information Structure on the Emergence of Differential Object Marking: An Experimental Study

**DOI:** 10.1111/cogs.13119

**Published:** 2022-03-16

**Authors:** Shira Tal, Kenny Smith, Jennifer Culbertson, Eitan Grossman, Inbal Arnon

**Affiliations:** ^1^ Department of Cognitive Sciences The Hebrew University of Jerusalem; ^2^ Centre for Language Evolution, School of Philosophy, Psychology and Language Sciences University of Edinburgh; ^3^ Department of Linguistics The Hebrew University of Jerusalem; ^4^ Department of Psychology The Hebrew University of Jerusalem

**Keywords:** Learning biases, Artificial language learning, Information structure, Language universals, Differential object marking

## Abstract

Many languages exhibit differential object marking (DOM), where only certain types of grammatical objects are marked with morphological cases. Traditionally, it has been claimed that DOM arises as a way to prevent ambiguity by marking objects that might otherwise be mistaken for subjects (e.g., animate objects). While some recent experimental work supports this account, research on language typology suggests at least one alternative hypothesis. In particular, DOM may instead arise as a way of marking objects that are atypical from the point of view of information structure. According to this account, rather than being marked to avoid ambiguity, objects are marked when they are given (already familiar in the discourse) rather than new. Here, we experimentally investigate this hypothesis using two artificial language learning experiments. We find that information structure impacts participants’ object marking, but in an indirect way: atypical information structure leads to a change in word order, which then triggers increased object marking. Interestingly, this staged process of change is compatible with documented cases of DOM emergence. We argue that this process is driven by two cognitive tendencies. First, a tendency to place discourse given information before new information, and second, a tendency to mark noncanonical word order. Taken together, our findings provide corroborating evidence for the role of information structure in the emergence of DOM systems.

## 1 Introduction

There are thousands of spoken and signed languages in the world today. Despite their great diversity (Evans & Levinson, 2009), languages also show structural commonalities. One of the central objectives of the language sciences is to explain what gives rise to such commonalities. Alongside historically contingent events and environmental factors, a variety of cognitive and communicative pressures have been proposed to impact how languages are structured and how they change over time (Bybee, 2007; Christiansen & Chater, 2008; Culbertson & Kirby, 2016; Gibson et al., 2019; Givón, 1991; Zipf, 1949). In recent years, new experimental paradigms, most prominently artificial language learning experiments, have been used to investigate the link between recurrent typological patterns and different cognitive biases (Culbertson et al., 2012, 2020; Fedzechkina et al., 2012, 2017; Hudson Kam & Newport, 2005; Kurumada & Grimm, 2019; Martin & Peperkamp, 2020; St. Clair et al., 2009). In one of the paradigms used, participants are exposed to input that contains some variation, and the extent to which their productions deviate from the input is taken to reflect their preferences (for detailed reviews, see Culbertson, 2012; Tily & Jaeger, 2011). In such experiments, participants are found to change their input throughout the learning process in ways that are compatible with cross‐linguistic tendencies, thereby allowing researchers to isolate the impact of learning biases on linguistic structures (e.g., Culbertson et al., 2012; Fedzechkina et al., 2012; Hudson Kam & Newport, 2009). In the present article, we employ an artificial language learning paradigm to test long‐debated hypotheses about the role of cognitive pressures in the emergence and development of differential object marketing (DOM). DOM is a well‐documented phenomenon across languages, where only a subset of direct objects (also known as P‐arguments in the typological literature, Witzlack‐Makarevich & Seržant, 2018) require additional grammatical marking, whereas others are left unmarked (Bossong, 1985; Iemmolo, 2010; Moravcsik, 1978; Witzlack‐Makarevich & Seržant, 2018). For example, in Spanish, human objects have to be accompanied by the accusative marker *a* (1a), whereas nonhuman objects do not (1b). Such situations are known as coding asymmetries (Haspelmath, 2021a).

(1) Spanish (Indo‐European, Spain; Haspelmath, 2021a: 10)
  a. veo**a**lamujer
    see.pres.1sg**acc**the.fsgwoman
   “I see the woman.”
  b. veolacasa
    see.pres.1sgthe.fsghouse
   “I see the house.”

**Fig. 1:**
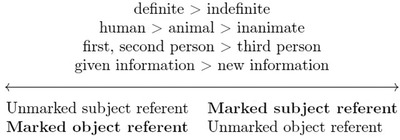
Referential scales illustrating various properties; those to the left are more typical of referents that appear in the subject position, whereas those to the right are more typical of referents in the object position.

Importantly, although not all direct objects are marked, the occurrence of marking is not random: it is typically impacted by semantic or discourse‐related properties of the direct object. For example, in languages like Spanish and Maltese, DOM is conditioned by the animacy of the object (Haspelmath, 2021b; Iemmolo, 2013). In Hebrew, DOM is conditioned by definiteness: only definite objects are marked (Haspelmath, 2021a). These coding asymmetries reveal an important generalization: direct objects are more likely to be marked when they have properties that typically characterize sentential subjects^1^ (known as S/A arguments in the typological literature, Witzlack‐Makarevich & Seržant, 2018). This phenomenon can be represented through referential scales, as shown in Fig. 1 (Croft, 2003). Values for properties toward the left part of the scale characterize referents that typically appear in the subject position (such as animate entities), whereas properties on the right side of the scale characterize referents that typically appear in the object position (such as inanimate entities). To give an example, a human definite entity is a typical subject but an atypical direct object.

A crucial insight of typological research is that atypical associations between referential properties and grammatical roles tend to receive more grammatical marking across languages (Bossong, 1991; Croft, 2003; Levshina, 2021; Silverstein, 1976). For example, if direct objects are typically inanimate, then utterances in which the direct object is animate show an atypical association or “mismatch” between grammatical roles and expected referential properties. This type of mismatch would then be expected to be marked more often—for example, by a case morpheme.

While DOM is found in many languages, there is a long‐standing debate about the mechanisms that cause such systems to develop and, in particular, what role specific cognitive or communicative pressures might play in the process. In what follows, we describe two prominent accounts for the emergence of DOM.

### 1.1 Ambiguity avoidance

It has often been suggested that DOM serves an *ambiguity avoidance function* (Bossong, 1985; Comrie, 1978; Dixon, 1994). In comprehending transitive sentences with two arguments, a key task for the listener is to understand which argument is the subject and which is the object. For example, for events where one argument is inanimate, such as one involving *Helena*, *door*, and *kicking*, assigning the likely grammatical roles to the referents (*Helena*, *door*) is unproblematic. As depicted in the scales in Fig. 1, because *door* is inanimate, it is less likely to be the subject of the sentence (and similarly, the animate entity *Helena* is unlikely to be the object). However, if the kicking in the previous example involves two animate entities, such as *Helena* and *Martha*, the listener may need additional cues to assign these entities to the subject and the object roles. English makes systematic use of word order to mark the distinction between subject and object (“Helena kicked Martha” vs. “Martha kicked Helena”), while other languages use different means, such as object marking (or other case marking, Bossong, 1985; Comrie, 1978; Dixon, 1994). From this perspective, DOM develops to avoid ambiguity, that is, to distinguish subjects from objects. Importantly however, it does so *efficiently*––objects are only marked when doing so helps reduce ambiguity (i.e., in cases where the two entities can serve both grammatical roles).

This ambiguity avoidance account fits within a communicative efficiency framework, according to which speakers strive to achieve an optimal balance between the pressure to minimize production effort and maximize understandability (Gibson et al., 2019; Levshina & Moran, 2021; Levy & Jaeger, 2007). Under the assumption that producing case marking is effortful, then a language adapted for communicative efficiency should restrict object marking to instances where it is needed to avoid ambiguity (Fedzechkina et al., 2012, 2017; Fedzechkina & Jaeger, 2020; Gibson et al., 2013; Jäger, 2007). This account has gained support from an artificial language learning study simulating the emergence of DOM (Fedzechkina et al., 2012, Experiment 1). In this study, participants were trained on a language used to describe events in which actions were performed by animate characters on either animate or inanimate patients. In other words, subjects were always animate, but objects could be either animate or inanimate. This language had flexible word order (60% SOV and 40% OSV in the initial input) and optional object case marking (60% of sentences). Importantly, the appearance of the object marker in the input was random and not conditioned on object type (animate vs. inanimate). This pattern of variable object marking does *not* follow the typical DOM pattern of marking atypical animate objects more frequently. The combination of flexible word order and random object marking yields a communicatively inefficient language: utterances with *animate* objects are ambiguous when they do not have object case marking, and utterances with *inanimate* objects are never ambiguous and yet sometimes have object case marking anyway.

Participants were trained on this language over the course of 4 days. At the end of days 2–4, participants were required to produce descriptions of events in the miniature language. Following the hypothesis that DOM is triggered by the tendency to disambiguate between subjects and objects, Fedzechkina et al. (2012) predicted that learners would introduce animacy‐contingent object marking to reduce ambiguity in the language. Specifically, while avoiding ambiguity could in principle be achieved by marking all objects, under the hypothesis that speakers are driven by a bias to avoid ambiguity *efficiently*, learners were instead predicted to selectively mark animate objects. This is indeed what they found: In producing the language themselves, learners diverged from their input by marking animate objects more frequently and marking inanimate objects less frequently.

Although Fedzechkina et al. (2012) argue that their study is evidence for the role of ambiguity avoidance in the emergence of DOM, this explanation has been challenged on several different fronts. First, cross‐linguistically, there appear to be very few languages where DOM can be said to be primarily conditioned by ambiguity avoidance, and even in these languages, this function tends to be overridden by other factors (Haspelmath, 2019, 2021b; Seržant, 2019). For example, it is common to find languages that allow the omission of marking in ambiguous contexts or that use object marking redundantly in unambiguous contexts (Haspelmath, 2021b; Seržant, 2019). In fact, an alternative interpretation of Fedzechkina et al. (2012) is that learners were producing redundant marking; although sentences with animate objects and no marking were considered ambiguous by Fedzechkina et al. (2012), in principle they were not. This is because in their design, specific animate characters were always either subjects or objects throughout the experiment (but not both). The fixed roles of the different animate characters could in principle serve as a consistent cue to their grammatical role without case marking. For example, if the chef character always appeared as a subject and never as an object, it could be interpreted as the subject regardless of word order and object marking. Using the Fedzechkina et al. (2012) paradigm, a recent study showed that participants do in fact use this lexical cue for sentence interpretation. Moreover, learning from an ambiguous input (where there were no fixed roles for the animate characters) had no effect on participants’ tendency to introduce DOM. This suggests that participants’ apparent restructuring of the input language is not necessarily driven by a bias to efficiently reduce ambiguity (Smith & Culbertson, 2020).^2^ Instead, Smith and Culbertson (2020) found that participants’ tendency to over‐ or undermark animate objects depended on the frequency of marking in the input, which is more consistent with a second explanation of DOM, which we call *predictability‐based marking* accounts. Finally, it is worth noting that the ambiguity avoidance explanation also fails to be clearly supported by diachronic evidence (Cristofaro, 2013, 2019). In particular, even when languages show patterns consistent with the ambiguity avoidance function, they seem to arise diachronically from a different source, as detailed below.

### 1.2 Predictability‐based marking

An alternative functional explanation for DOM is that DOM is a subcase of the more general phenomenon of *predictability‐based marking* where languages mark less expected forms with more linguistic material (Haspelmath, 2019, 2021b, 2021a; Levshina, 2021). According to this account, atypical associations between a referent and the grammatical role it is assigned are marked due to their atypicality, regardless of ambiguity.^3^ In the case of animacy‐conditioned DOM, since animate referents appear more frequently in the subject role than in the object role, animate objects tend to receive more marking crosslinguistically. That is, increased marking of animate objects can be explained without resorting to ambiguity avoidance. The predictability‐based marking account is compatible with the communicative efficiency framework: Assigning more linguistic form (in this case, object marking) to less predictable meanings (e.g., animate objects) is communicatively efficient (Gibson et al., 2019; Kanwal et al., 2017; Kurumada & Jaeger, 2015; Pate & Goldwater, 2015; specifically on DOM see Haspelmath, 2021a, 2021b; Levshina, 2021). Notably, as seen in Fig. 1, the association between referents and grammatical roles can be atypical in various ways, including pragmatic discourse properties of *topicality* and *givenness*. Topical information refers to backgrounded or assumed information. Topical elements are usually *given*, referring to information previously known or discussed (Arnold et al., 2013; Chafe, 1976; Iemmolo, 2010).^4^ While given/topical (old) information typically appears in the subject role, new information tends to appear in the object role (Du Bois, 1987; Haspelmath, 2021b; Levshina, 2021).^5^ These properties pertain to the linguistic domain of *information structure*: the relation between the propositional content of an utterance and the addressee's state of knowledge at the time of utterance (Arnold, 2016; Dalrymple & Nikolaeva, 2011). If information structure mismatches also impact marking—in addition to fixed semantic properties, such as animacy—then given/topical objects should be marked with more linguistic material.

In line with this prediction, there are many documented cases of DOM triggered by information structure, in which unexpected given/topical objects receive special case marking (e.g., Tigre, Persian, Balearic Catalan, and Altai; Dalrymple & Nikolaeva, 2011; Escandell‐Vidal, 2009; Iemmolo, 2010, 2011, 2013). For example, in Tigre (a Semitic language), objects that are introduced into the discourse for the first time (i.e., are informationally new) are unmarked (2a), whereas discourse‐old objects are marked (2b). Note that this is regardless of the fact that in both cases, the object is animate and definite (Dalrymple & Nikolaeva, 2011).

(2) Tigre (Semitic, Eritrea; Raz, 1983: 104–109)
  a. giswagabilyemənˀədefarˀonˀafgər
    goand.my.peoplefromhandPharaohbring.out
   “Go and free my people from the hands of Pharaoh.”
  b. (after introducing the cat and the dog):
    daˀamdəmmu…**ˀəgəl**kaləbwəˀultalmatˀəttu
    butcat**Prep**dogdeliberatelyshe.deceivedhim
   “But the cat … deliberately deceived the dog.”

Importantly, this is not a rare pattern cross‐linguistically: Out of a sample of 133 languages with DOM, in 64% of them, DOM was primarily triggered by information structure (Iemmolo, 2011, 2013). Moreover, it has been suggested that even DOM systems that are synchronically governed by semantic properties of the object originate from atypical information structure (Cristofaro, 2013, 2019; Dalrymple & Nikolaeva, 2011; Iemmolo, 2010; König, 2008). Critically, the reason for this can be traced back to the scales in Fig. 1: given/topical referents also tend to be animate and definite (Cristofaro, 2013, 2019; Givón, 1976; Iemmolo, 2010; Levshina, 2021). Under this account, the close association between information structure and semantic properties leads to the eventual loss of the link between DOM and information structure; the marking is reanalyzed as being conditioned by fixed properties, such as animacy (Cristofaro, 2013, 2019; Dalrymple & Nikolaeva, 2011; Iemmolo, 2010, 2013). This diachronic chain of events can be seen, for example, in Sicilian (Iemmolo, 2010) and Chichewa (Downing, 2018).

### 1.3 Summary

The existing literature provides us with two different explanations for the emergence of DOM systems in language: ambiguity avoidance and predictability‐based marking of atypical associations. The former account often highlights the special role of fixed semantic properties, such as animacy; the latter emphasizes the role of multiple factors, including context‐dependent discourse‐pragmatic properties, such as information structure.^6^ In the present paper, we use artificial language learning experiments to explore the relation between DOM and information structure in an attempt to provide experimental support for predictability‐based marking accounts of DOM. Specifically, we report two artificial language learning experiments, both modeled after Fedzechkina et al. (2012): participants were trained on a language with object marking that was optional and unconditioned on any other property of the language. We introduce a novel information structure manipulation by making the object of each sentence either given or new. Unlike in Fedzechkina et al. (2012), we use only animate objects. If DOM is driven primarily by ambiguity avoidance, then the use of marking should not be influenced by information structure status (given vs. new). Alternatively, if DOM can emerge via predictability‐based marking, then (atypical) given objects should trigger more marking than (typical) new objects. We test these predictions in two experiments.

In Experiment 1, word order in the input language was variable (as in Fedzechkina et al. 2012); therefore, sentences without object marking were always ambiguous. To preview, we found that participants were not more likely to use the object marker on atypical given objects. However, we found an *indirect* effect of information structure on object marking: Information structure impacted participants’ choice of word order––leading to an increase in OSV order when the object was given––and this OSV order resulted in increased marking of the object. In Experiment 2, we sought to further explore the direct relation between information structure and object marking that is predicted by the predictability‐based marking account (Haspelmath, 2019, 2021b, 2021a; Levshina, 2021). In Experiment 2, word order was fixed, so participants would not condition it on information structure (or any other property of the language); information structure could only affect object marking directly. Here, we again failed to find an influence of information structure on object marking. Our findings highlight the impact of other factors beyond ambiguity on DOM and suggest that, at least in cases where atypical information structure triggers DOM, word order may play an important role in its emergence (Iemmolo, 2010, 2013).

## 2 Experiment 1

The experiment was modeled after Fedzechkina et al. (2012), using the materials and design of Smith and Culbertson (2020), who replicated Fedzechkina et al. (2012) online.

### 2.1 Method

#### 2.1.1 Participants

Participants were recruited through Amazon Mechanical Turk (MTurk). They were self‐reported native speakers of English aged 18 or over. Any English‐speaking participant based in the United States was qualified to participate on the first day of the experiment. Only those who satisfied the progression criterion (detailed below) could continue to the second day. Participants were paid either $4 or $6 for each day of the experiment (detailed below). The total number of participants on each day was as follows: day 1: 85; day 2: 63; day 3: 51; and day 4: 45. The number of participants declined each day due to participants who did not satisfy the progression criterion (see below) or participants who did not return on all four days. Only participants who completed the entire four days of the task were included in the analyses: this resulted in 43 participants (two participants started day 4 but did not complete it). Importantly, the pattern of results did not change when all participants were included.

#### 2.1.2 Input language and stimuli

The artificial language contained four verbs and eight nouns. All of the nouns referred to animate characters. There were 15 possible labels for nouns, eight of which were randomly selected for each participant (see Table 1 for full language lexicon). The language had flexible word order: 50% SOV and 50% OSV,^7^ that is, the verb always came at the end of the sentence, the subject noun was before the object noun in half of the training sentences, and after the object in half of the sentences. In addition, the language had optional object marking (a suffix, *‐ka*, that was attached to the noun) that appeared in 50% of the sentences. The object marking was not conditioned by any property of the language: it appeared equally often with both word orders and for both types of objects (given and new, detailed in the next section), and it was not conditioned on the identity of the verbs or nouns. Subjects were never marked.

**Table 1:** Full lexicon of the artificial language

Nouns		Verbs	Object marker
slagum	tombat	slergin	‐ka
nagid	melnog	prog	
norg	daf	shen	
plid	klamen	zamper	
dacin	zub		
vams	bliffen		
rungmat	lombur		
groost			

Note: These labels were taken from Fedzechkina et al. (2012).

The language was presented to participants both as text and auditorily. Sound files were generated using the Tessa voice in the MacTalk speech synthesizer, with pitch and tempo increased by 30% using Audacity (this was done in order to suit the character of the tutor who trained participants on the language––a friendly monster).

The sentences described drawings depicting simple transitive events in which animate characters performed actions on other animate characters.^8^ The events included four possible actions (kicking, punching, shooting, and touching) and 10 possible characters (artist, boxer, burglar, chef, clown, cowboy, dancer, medic, police officer, and waiter). Of these 10 characters, eight were randomly selected for each participant. The assignment of labels to characters and actions was random for each participant. This procedure ensured that any potential nonarbitrary associations between characters, actions, and vocabulary items could not systematically bias our results.

#### 2.1.3 Manipulation of information structure

**Fig. 2:**
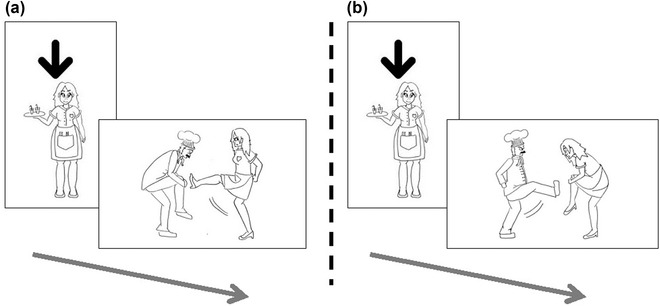
An example trial of (A) a typical alignment (given subject, new object) and (B) an atypical alignment (given object, new subject).

The sentences presented to participants were typical or atypical with respect to information structure. We manipulated the information structure by making either the subject or the object of each sentence more given/topical in the following way. Before each sentence, one of the characters was presented on screen alone for 2 seconds with an arrow pointing to them (Fig. 2). After this, participants heard a transitive sentence in which this character turned out to be either the doer of the action (subject) or the character the action was done to (object). In 50% of the sentences, the introduced character turned out to be the subject of the following sentence (e.g., *waitress…the waitress kicked the chef*), thereby creating a *typical* sentence with respect to information structure,^9^ with given information appearing in the subject position. In the other 50% of the sentences, the character turned out to be the object of the following sentence (e.g., *waitress…the chef kicked the waitress*), thereby creating an *atypical* sentence with respect to information structure, with given information appearing in the object position.

#### 2.1.4 Procedure

The procedure was adopted from Smith and Culbertson (2020). Participants were informed that they would learn an alien language called Smeespeak, taught by a monster named Smeeble. Throughout the experiment, Smeeble appeared on the right part of the screen and gave feedback during certain learning stages (see below). Each of the four days of the experiment included all phases detailed below, except for the first day in which there was no *sentence production test* (matching the procedure in Fedzechkina et al. 2012). Participants were paid $6 for each day, unless they did not pass the second *noun comprehension test* (see below)––in which case they stopped the experiment half‐way, were paid only $4, and did not qualify for the next day of the experiment. Participants who did not pass the *sentence comprehension test* (see below) were also not qualified to continue to the next day of the experiment but were paid $6.

**Fig. 3:**
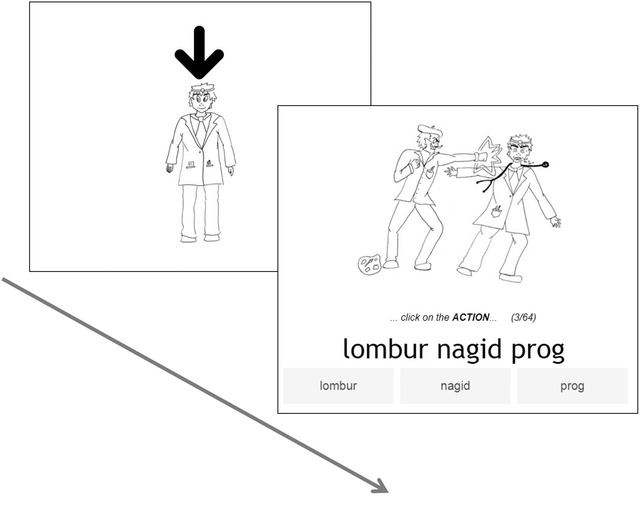
An example trial in the sentence training phase. In this example, the character introduced in the pretrial image is the object of the following sentence, rendering the sentence atypical in terms of information structure (given information appears in the object role).

**Fig. 4:**
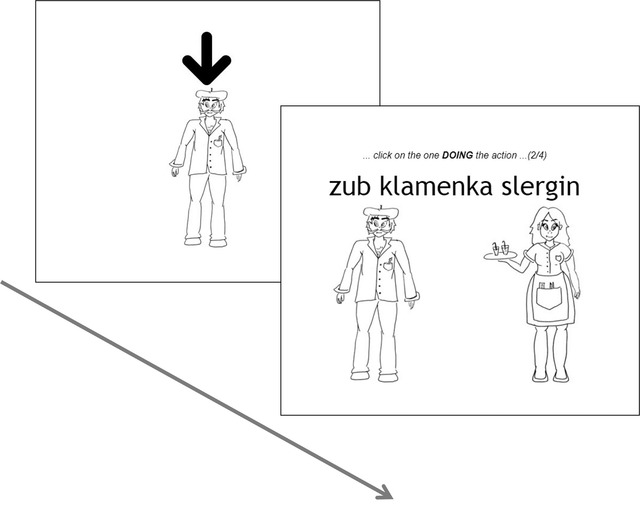
An example trial in the sentence comprehension test phase.

**Fig. 5:**
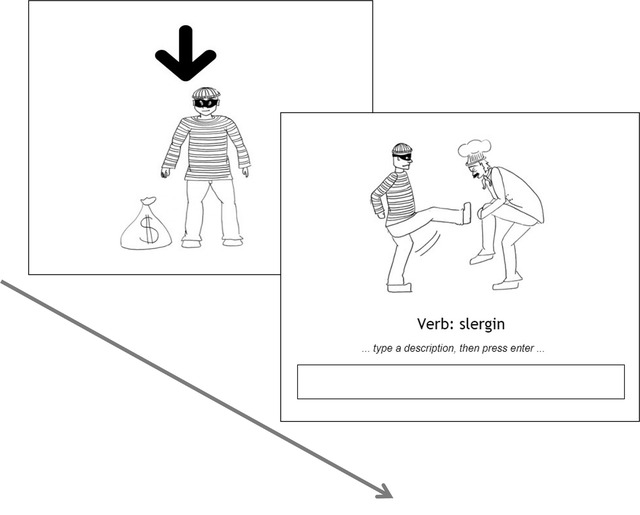
An example trial in the sentence production test phase. In this example, the character introduced in the pretrial image is the subject of the following sentence, rendering the sentence typical in terms of information structure (given information appears in the subject role).

1. *Noun training*: In each trial, participants were presented with a drawing of a character accompanied by its name in the novel language. Alongside the character, two buttons appeared onscreen: one showed the character's correct label (that was just heard), and another showed a randomly selected label. Participants had to click on the correct label. Feedback was provided after each trial, and trials where participants clicked on the wrong labels were repeated until they were correct. Participants received 16 such trials (two trials per character).
2. *Noun comprehension test*: In each noun comprehension trial, participants saw two characters and heard one noun (auditorily and in written form). Participants had to choose the character that matched the label. Feedback was provided after each trial (correct responses resulted in a success sound, a happy‐looking Smeeble, and the addition of 10 points; incorrect responses resulted in a failure sound, a sad‐looking Smeeble, and no points). Participants progressed to the next trial regardless of their success or failure. Participants received eight such trials (one trial per character).
3. *Sentence training*: In each trial, participants first saw one of the characters (with an arrow on top, see 2.1.3 and Fig. 2) without any accompanying label. They then saw a drawing of a transitive action, involving the previously introduced character and another character, and heard its audio and text description in Smeespeak. Under the written sentence, there were three buttons corresponding to each of the words (the subject, the object, and the verb, see Fig. 3). The object label always appeared without marking regardless of whether the sentence had object marking. The labels appeared in the same order as they appeared in the sentence (e.g., for an SOV sentence, the order of the buttons was subject, object, and verb). In each trial, participants were instructed to complete one of the following instructions (randomly chosen): “click on the one DOING the action,” “click on the one the action is DONE TO,” or “click on the ACTION” (see Fig. 3 for an example). Feedback was provided after each trial. Trials where participants clicked on the wrong button were repeated until they were correct. Participants received 64 such trials.
4. *Noun training*: Same procedure as the first noun training phase. Before the first trial, participants were informed that they would be trained on the character names again, but this time they were required to pass the subsequent comprehension test to continue the experiment. Participants received 16 such trials (two trials per character).
5. *Noun comprehension test*: The same procedure as the first noun comprehension phase; however, as mentioned above, this test determined progression in the experiment. Participants had to score at least 75% correct in order to progress to the next stage of the experiment and qualify for the next day. If they did not reach this criterion, they were paid $4 for their participation up to this point. Participants received eight such trials (one trial per character).
6. *Sentence comprehension test*: Before this phase started, participants were again informed that further progression in the experiment would depend on satisfactory performance. Similar to the sentence training phase described above, each trial began with an image introducing a character, followed by a sentence involving that character. However, in this phase, the sentence was accompanied by two images, one of each character in the sentence. Their instruction was always to “click on the one DOING the action” (see Fig. 4 for an example). As in noun comprehension trials, participants received feedback on their response. On the first day of the experiment, this was the final phase, and all participants qualified (regardless of their performance accuracy). On days 2–4, however, participants who scored below 50% correct during this test did not progress to the next stage of the experiment and did not qualify for the next day. Participants received 64 such trials.
7. *Sentence production test*: This phase appeared on days 2–4 only. As in the sentence training phase, in each trial, participants first saw a character introduced, and then an image depicting a transitive action involving the previously introduced character and another character. This time, however, participants had to describe the image in Smeespeak themselves by typing into a text box. Following Fedzechkina et al (2012), participants were provided with the appropriate verb for each trial (see Fig. 5 for an example). Participants received 32 such trials.

#### 2.1.5 Scoring

Participants’ word order (SOV or OSV) and use of object marking (present or absent) were automatically scored, adopting the procedure in Smith and Culbertson (2020). Each typed description was broken into words bounded by whitespace. For each word, the closest matching label from the trained vocabulary was identified, allowing for the object marker to be attached to any of the words. Following previous studies (Fedzechkina et al., 2012; Smith & Culbertson, 2020), we only included sentences allowed by the training language: ones which had the correct characters, used SOV or OSV order, and attached the marker (if present) to the object noun. All other trials were excluded from further analysis (i.e., trials in which one of the nouns did not correspond to any of the characters in the event; the word order used was not SOV or OSV; the object marker appeared on the subject or the verb). This resulted in the exclusion of 13% of the sentences and, as a byproduct, the exclusion of one participant (who produced only ungrammatical sentences).

### 2.2 Results

**Fig. 6:**
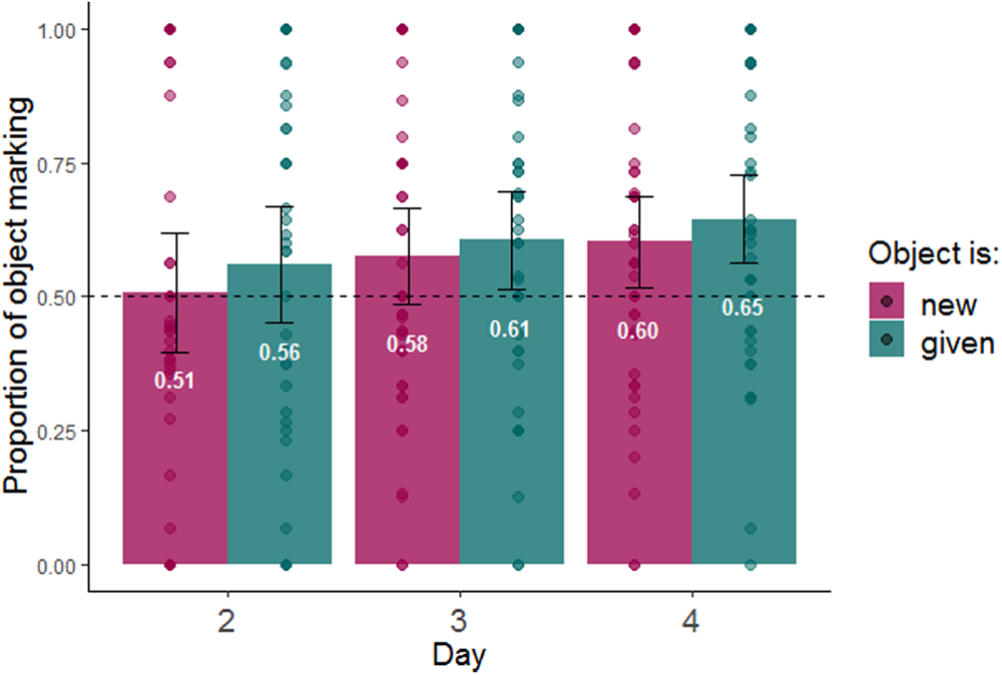
Proportion of object marking by information structure status of the objects during production testing (NB: testing occurred on days 2–4 only). The dashed line indicates the frequency of object marking in the input; error bars indicate confidence intervals of 95%; individual points indicate by participant means.

Fig. 6 shows the proportion of object marking as a function of the objects’ information structure status throughout the days. As seen in the plot, participants used more object marking for given rather than new objects.

**Fig. 7:**
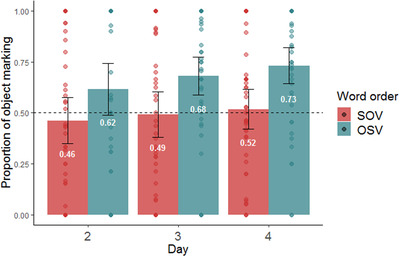
Proportion of object marking by word order during production testing (NB: testing occurred on days 2–4 only). The dashed line indicates the frequency of object marking in the input; error bars indicate confidence intervals of 95%; individual points indicate by participant means.

**Fig. 8:**
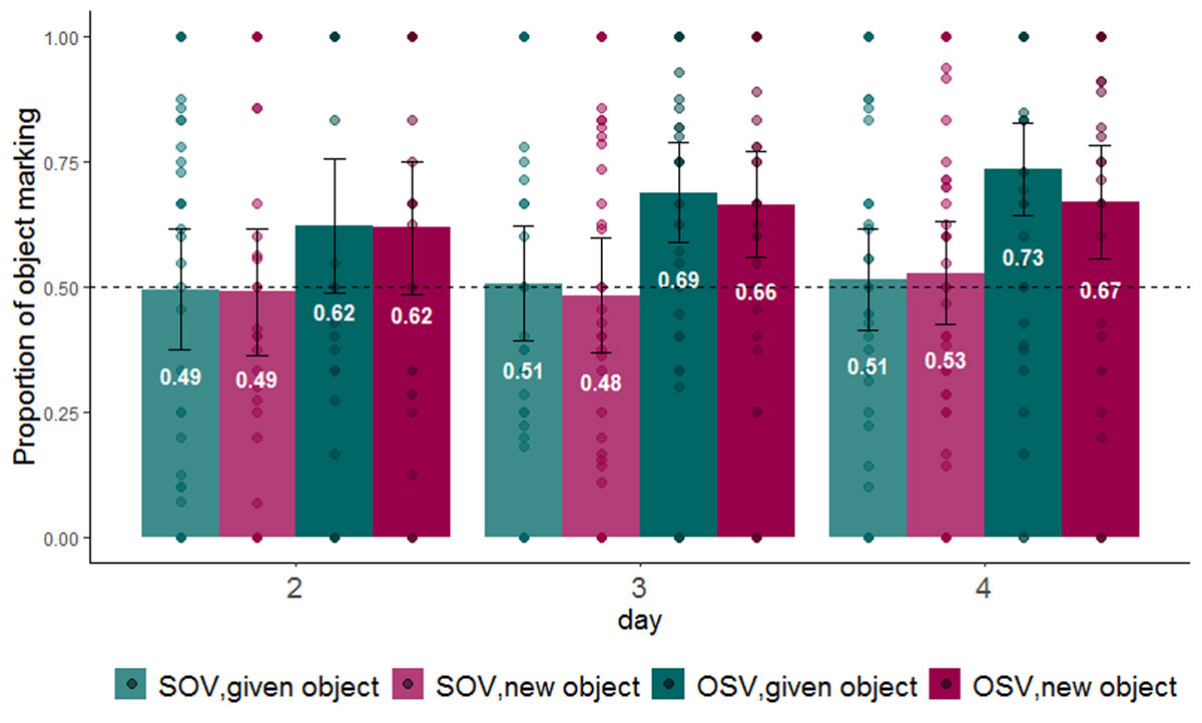
Proportion of object marking by word order and information structure during production testing (darker shades represent OSV). The dashed line indicates the frequency of object marking in the input; error bars indicate confidence intervals of 95%; individual points indicate by participant means.

We used a mixed‐effect logistic regression model to examine the effect of our information structure manipulation on participants’ use of object marking (here and in all subsequent models, we used the lme4 package in R software, Bates, Mächler, Bolker, & Walker, 2015, and the maximum random effect structure justified by the data that converged, Barr, Levy, Scheepers, & Tily, 2013). If object marking is impacted by information structure, speakers should be more likely to use object marking for given objects compared to new objects. The dependent variable was the use of object marking in each production trial. The model included fixed effects for information structure status (given vs. new, sum coded), day of training (2–4, sum coded), word order (SOV vs. OSV, sum coded), as well as all interactions between them. The model included random intercepts for participants and by‐participant random slopes for information structure status (see Table 2 for full model). In contrast to our prediction, we found no significant effect of information structure on object marking (β = 0.077, SE = 0.059, *p =* .191). We did, however, find an impact of word order on object marking. As seen in Fig. 7, participants were more likely to mark objects if the word order they produced was OSV (β = 0.472, SE = 0.046, *p*<.001). Interestingly, this is a repeated finding in previous literature (Fedzechkina et al., 2012, 2017; Fedzechkina & Jaeger, 2020; Smith & Culbertson, 2020). We discuss the possible reasons for this tendency in the General discussion. See Fig. 8 for the use of object marking as a function of both word order and information structure. Finally, we found a significant increase in object marking on day 4.

**Table 2:** Summary of the regression model of participants’ object marking productions in Experiment 1

	Estimate	Std. error	*z*‐value	*p*‐value
Intercept	0.655	0.286	2.293	.022 *
IS status = given	0.077	0.059	1.306	.191
Word order = OSV	0.472	0.046	10.325	< .001 ***
Day = 3	0.035	0.06	0.593	.553
Day = 4	0.216	0.061	3.569	< .001 ***
IS status = given * Word order = OSV	0.044	0.045	0.98	.327
IS status = given * Day = 3	−0.06	0.059	−1.01	.313
IS status = given * Day = 4	−0.017	0.06	−0.288	.774
Word order = OSV * Day = 3	0.103	0.06	1.709	.088.
Word order = OSV * Day = 4	0.045	0.061	0.739	.46
IS status = given * Word order = OSV * Day = 3	−0.042	0.06	−0.696	.486
IS status = given * Word order = OSV * Day = 4	0.064	0.06	1.052	.293

Note: *p < .05; **p < .01; ***p < .001.

**Fig. 9:**
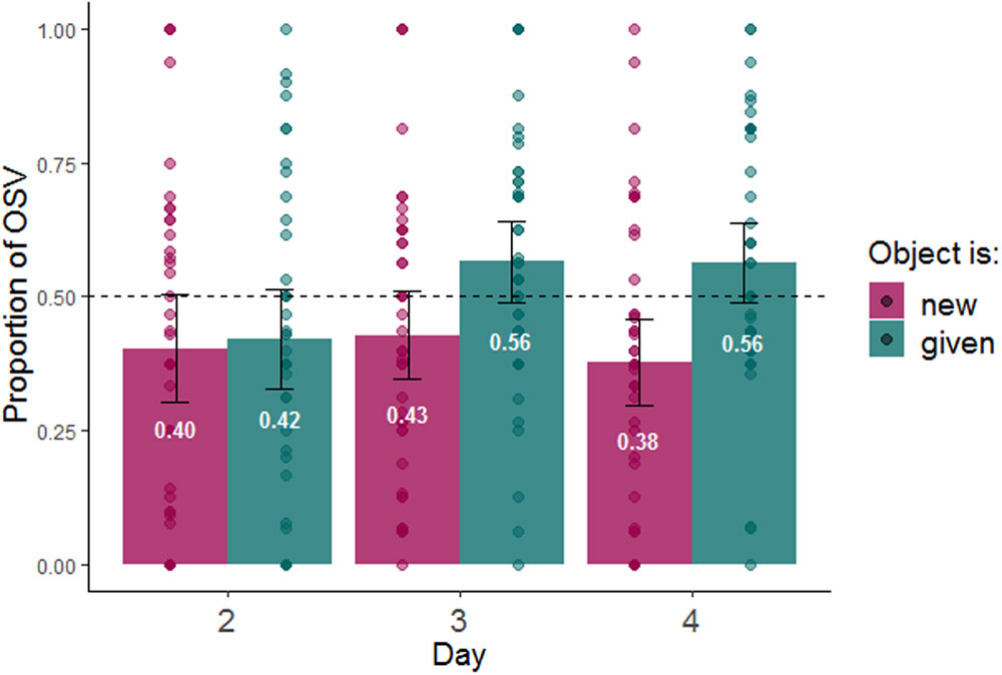
Proportion of OSV by information structure status of the objects during production testing (days 2–4 only). The dashed line indicates the frequency of OSV in the input; error bars indicate confidence intervals of 95%; individual points indicate by participant means.

Taken together, our information structure manipulation did not affect object marking. In contrast with the prediction of predictability‐based explanations for the emergence of DOM, given objects (an atypical association) were not marked more than new objects (a typical association). Rather, word order was the primary driver of case marking; when participants used OSV order, they were more likely to mark objects. However, both psycholinguistic and cross‐linguistic evidence suggest that word order is itself influenced by information structure (Arnold et al., 2013; Birner & Ward, 2009; Tomlin, 1995). Fig. 9 shows the proportion use of OSV as a function of the information structure status of the object across testing days in our study. This plot suggests that indeed, information structure impacted word order choice in our results as well: OSV was used more frequently when objects were given rather than new. We used a mixed‐effect logistic regression model predicting the use of OSV order (vs. SOV) from information structure status (given vs. new, sum coded), object marker use (marked objects vs. unmarked objects, sum coded), and day of training (2–4, sum coded), as well as all interactions between them. The model included random intercepts for participants and by participant random slopes for information structure status and object marking (see Table 3 for full model). The effect of information structure on word order was confirmed by our analysis: participants were significantly more likely to use OSV order when the object was given (β = 0.267, SE = 0.096, *p =* .005).

**Table 3:** Summary of the regression model of participants’ OSV productions in Experiment 1

	Estimate	Std. error	*z*‐value	*p*‐value
Intercept	−0.439	0.153	−2.869	.004 **
IS status = given	0.267	0.096	2.783	.005 **
Object marking = TRUE	0.594	0.163	3.654	< .001 ***
Day = 3	0.214	0.059	3.59	< .001 ***
Day = 4	0.051	0.061	0.847	.397
IS status = given * Object marking = TRUE	0.074	0.048	1.54	.124
IS status = given * Day = 3	0.057	0.059	0.961	.337
IS status = given * Day = 4	0.146	0.059	2.462	.014 *
Object marking = TRUE * Day = 3	0.045	0.06	0.761	.447
Object marking = TRUE * Day = 4	−0.021	0.061	−0.346	.73
IS status = given * Object marking = TRUE * Day = 3	−0.084	0.059	−1.419	.156
IS status = given * Object marking = TRUE * Day = 4	0.057	0.06	0.952	.341

In other words, in trials where the introduced character turned out to be the object of the following sentence (e.g., *waitress…the chef kicked the waitress*, Fig. 2B), participants tended to begin the sentence with the object. In contrast, in trials where this character turned out to be the subject of the following sentence (e.g., *waitress…the waitress kicked the chef*, Fig. 2A), participants preferred to begin their description with the subject. As mentioned above, information structure has been found to affect word order in natural languages as well, and in fact in just this way. Specifically, the choice to begin the sentence with the given element regardless of its syntactic role is in line with a well‐documented tendency in natural language to place given information before new information (Arnold et al., 2013, 2000; Clark & Clark, 1977; Gundel, 1988; Tomlin, 1995). We discuss this further in the next section. In addition, we found a significant effect of object marking on word order, such that OSV was likelier when object marking was used (β = 0.594, SE = 0.163, *p*<.001). There was also a significant increase in OSV on the third day of the experiment (compared with the grand mean, day 2: 41%, day 3: 50%, day 4: 47%, β = 0.214, SE = 0.059, *p*<.001). Finally, we found an interaction between information structure and day of training, such that on the final day of training (day 4), participants showed an increased preference for condition word order on information structure (β = 0.146, SE = 0.059, *p =* .014).

### 2.3 Discussion

In Experiment 1, we tested the prediction, based on predictability‐based accounts of the emergence of DOM, that atypical information structure would give rise to increased object marking for given objects. This prediction was not borne out. At first glance, this could suggest that our manipulation did not succeed in creating differences in information structure. Importantly, however, our manipulation *did* impact participants’ output, just not their use of object marking: while given objects were (numerically) marked more than new objects, this effect was driven by the impact of information structure on *word order*, not object marking itself. In other words, information structure *did* impact participants’ object marking, but in an indirect way, through their choice of word order. Our findings may reflect two separate biases that together bring about increased object marking. The first is the tendency to put given information before new information. Participants used OSV more than SOV when the object was given, mirroring a well‐established preference to put given information before new in natural languages (Arnold et al., 2013, 2000; Birner & Ward, 2009; Clark & Clark, 1977; Gundel, 1988; Kaiser & Trueswell, 2004; Tomlin, 1995). For example, although the canonical word order in Finnish is SVO, Finnish speakers use object‐first word order when the object is given and the subject is new (Kaiser & Trueswell, 2004; Kay & Karttunen, 1984). This pattern is exactly what we find in Experiment 1: although participants start out with a general preference for SOV sentences, they increase their use of OSV when the object is given. In other words, participants preferred to start their sentences with the given character, whether it was the subject (thereby creating SOV sentences) or the object (thereby creating OSV sentences). The second tendency we found was for object marking to be influenced by word order. As in previous artificial language learning studies (Fedzechkina et al., 2012, 2017; Fedzechkina & Jaeger, 2020), we found that participants used object marking more in OSV sentences than in SOV sentences. We elaborate on the possible reasons for this apparent bias in the General discussion.

Taken together, these results paint the following picture: information structure leads to an increased use of OSV word order, which in turn leads to more object marking. Importantly, this two‐step account, underpinned by the two individual biases—given‐before‐new and marking of OSV—presents another route to the emergence of DOM, an alternative to ambiguity avoidance. This word‐order‐mediated relationship between information structure and case marking is not the emergence path predicted by predictability‐based accounts of DOM (Haspelmath, 2019, 2021b, 2021a; Levshina, 2021). According to these accounts, given objects are atypical with respect to information structure, and should, therefore, receive more marking regardless of word order. Instead, our results are in line with typological data on the diachronic emergence of DOM systems from information structure (Downing, 2018; Iemmolo, 2010, 2013). In many languages, atypical information structure is reflected in a change of word order, such that objects appear in a position where typically given/topical information appears, instead of their canonical position (Austin, 2001; Escandell‐Vidal, 2009; Payne, 1992). In several documented languages, these topicalized objects then tend to be case marked (Escandell‐Vidal, 2009; Iemmolo, 2010, 2013). During this stage, then, DOM appears only in topicalized or dislocated objects. In other words, it is restricted to given objects in certain atypical word orders. This phenomenon is quite frequent cross‐linguistically: In a sample of 85 spoken languages where information structure is the main triggering function for DOM, 67% of the languages exhibit marking that is restricted to dislocated or topicalized objects (Iemmolo, 2011, 2013). In Chepang (Tibeto‐Burman), for example, when the default (or basic) SOV word order is used, the object is not marked (3a). However, when the direct object is topicalized and appears in a noncanonical sentence‐initial position, it receives marking (3b).

(3) Chepang (Tibeto‐Burman, Nepal; Caughley, 1982: 68)
*a*. *Puʔ‐nis‐ʔi* *həw* *sat‐ʔaka‐c‐u*
    older_brother‐du‐ergyounger_brotherkill‐pst‐du‐a
   “The two older brothers killed the younger brother.”
*b*. *həw‐* ***kay*** *puʔ‐nis‐ʔi*sat‐*ʔ*a‐th*ə*y
    younger_brother‐**ACC**older_brother‐DU‐Akill‐PST‐OBJ
   “The two older brothers killed the younger brother.”

To summarize, the results of Experiment 1 are compatible with a proposed diachronic pathway in which atypical information structure is reflected in word order, which in turn leads to increased marking of objects in noncanonical sequential positions (in our experiment, OSV word order). However, it is possible that our failure to find a clear effect of information structure on object marking was driven by the variable word order; if the given‐before‐new bias is stronger than the bias to mark atypical objects, it might have masked the direct effect of information structure on object marking in our design (participants may prefer to change word order rather than increase object marking). To test this, we ran Experiment 2, which was identical to Experiment 1 except that word order was fixed and, therefore, could not be used to mark information structure. If atypical information structure‐grammatical role pairings directly give rise to DOM, then we should see increased object marking for given objects in this design.

## 3 Experiment 2

In Experiment 2, we ask whether participants will create an information structure‐based DOM system when word order in the input language is fixed, and thus, the only aspect of the language that can be conditioned on information structure is case marking. We used the same paradigm as in Experiment 1, but with an input language in which SOV word order is used 100% of the time. If we find that under these conditions, given objects are marked more compared to new objects, this would provide support for a direct link between information structure and DOM emergence (Haspelmath, 2019, 2021b, 2021a; Levshina, 2021). If, as in Experiment 1, object marking is not impacted (directly) by information structure, this would instead support a staged account, where dislocation of the given object (i.e., change in word order) has a mediating role in the emergence of DOM (Downing, 2018; Iemmolo, 2010, 2013).

### 3.1 Method

#### 3.1.1 Participants

Participants were recruited and paid in the same way as in Experiment 1. The total number of participants on each day was as follows: day 1: 56; day 2: 50; day 3: 45; and day 4: 44. Again, only participants who completed the entire four days of the task were included in the analyses: this resulted in 43 participants (one participant started day 4 but did not complete it). Importantly, the pattern of the results did not change when all participants were included.

#### 3.1.2 Input language and stimuli

The stimuli and language were identical to those used in Experiment 1, with two notable differences. First, the language had a fixed word order: 100% SOV. Second, the optional object marking now appeared in 62.5% of the sentences.^10^

#### 3.1.3 Manipulation of information structure

Same as Experiment 1.

#### 3.1.4 Procedure

The procedure was identical to Experiment 1, except for a slight change in the progression criteria: since the language used in this experiment was easier to learn compared to Experiment 1 (only one word order, and as a result no possible ambiguity), the progression criterion in the sentence comprehension phase was increased to 70% on all days.

#### 3.1.5 Scoring

The same procedure as in Experiment 1. Again, we excluded ungrammatical productions from our analysis: This resulted in the exclusion of 20% of the sentences, and as a byproduct the exclusion of four participants who produced only ungrammatical sentences (remaining *n* = 39).

## 4 Results

**Fig. 10:**
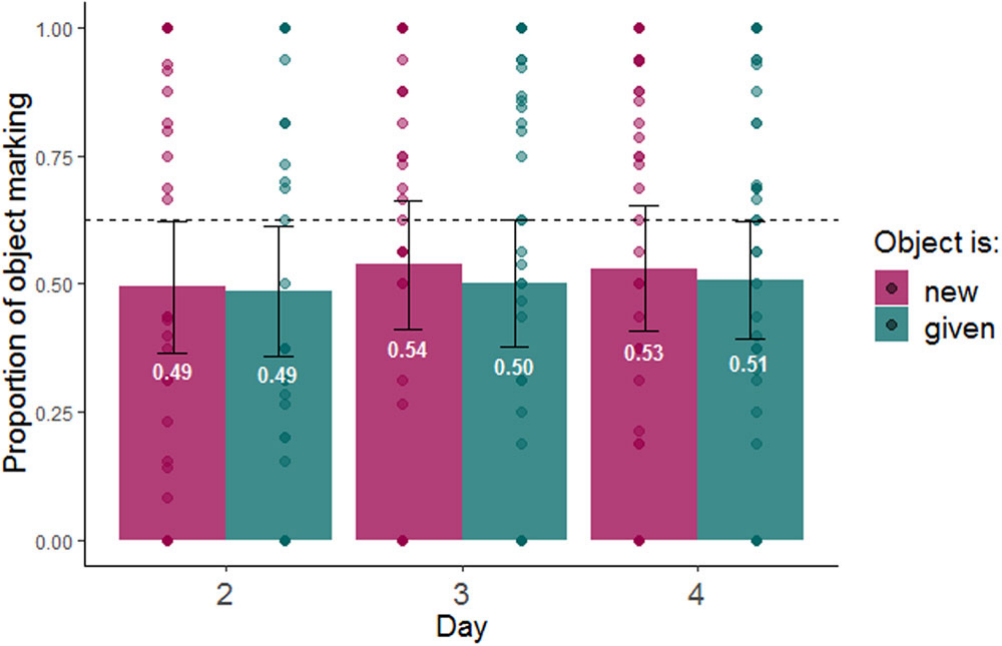
Proportion of object marking by information structure status of the objects during production testing (days 2–4 only). The dashed line indicates the frequency of object marking in the input (62.5%); error bars indicate confidence intervals of 95%; individual points indicate by participant means.

Fig. 10 shows the proportion of object marking as a function of the information structure status of the object. The slight trend evident in the plot does not appear to support the predictability‐based marking account: participants do not mark given objects more than new objects; in fact, the trend is in the opposite direction. We used a mixed‐effect logistic regression model predicting the use of object marking in each production trial from information structure status (given vs. new, sum coded) and day of training (2–4, sum coded), as well as the interaction between them. The model also included random intercepts for participants (see Table 4 for full model). Information structure did not impact object marking (β = –0.08, SE = 0.046, *p =* .083). We also found increased object marking on day 3 of the experiment (compared with the grand mean, β = 0.131, SE = 0.066, *p =* .047).

**Table 4:** Summary of the regression model of participants’ object marking productions in Experiment 2

	Estimate	Std. error	*z*‐value	*p*‐value
Intercept	−0.194	0.471	−0.411	.681
IS status = given	−0.08	0.046	−1.732	.083.
Day = 3	0.131	0.066	1.988	.047 *
Day = 4	−0.025	0.065	−0.38	.704
IS status = given * Day = 3	−0.063	0.066	−0.968	.333
IS status = given * Day = 4	−0.002	0.064	−0.033	.973

### 4.1 Discussion

Experiment 2 set out to ask whether atypical information structure has a direct impact on object marking, as predicted by the predictability‐based marking account (Haspelmath, 2019, 2021b, 2021a; Levshina, 2021) We found that, as in Experiment 1, and in contrast with the predictability‐based marking account, information structure did *not* impact object marking directly. To sum up the results, in Experiment 1, we found that information structure affected word order and that word order affected object marking. However, across both experiments, we found no evidence for a direct relation between atypical information structure and object marking. These findings are most consistent with the idea that the impact of information structure on DOM is mediated by changes in word order: atypical information structure leads to a change in word order, which is then followed by increased object marking (Downing, 2018; Escandell‐Vidal, 2009; Iemmolo, 2010, 2013).

## 5 General discussion

The function of DOM and the mechanism by which it emerges have been a central topic of research and debate among linguists and, more recently, cognitive scientists. Here, we focused on two competing accounts. According to the first, DOM systems emerge due to a pressure for ambiguity avoidance—speakers’ need to disambiguate between subjects and objects (Bossong, 1985; Comrie, 1978; Dixon, 1994). This account has recently gained support from artificial language learning studies (Fedzechkina et al., 2012; though see Smith & Culbertson, 2020 for an alternative account). However, recent diachronic and crosslinguistic research questions whether ambiguity avoidance is in fact the driving force behind the emergence of DOM systems (Cristofaro, 2013, 2019; Haspelmath, 2019; Iemmolo, 2010). According to an alternative account, unpredictable or atypical alignments of event participants and roles—for example, objects that are animate or given—receive marking due to their atypicality, regardless of actual or potential ambiguity. This predictability‐based marking account highlights the role of atypical information structure in the emergence of DOM systems (Cristofaro, 2013, 2019; Dalrymple & Nikolaeva, 2011; Iemmolo, 2010, 2013; König, 2008).

Here, we set out to investigate whether atypical information structure drives the emergence of DOM when ambiguity is not an issue (because all sentences are potentially ambiguous). We investigated this in two artificial language learning experiments modeled after Fedzechkina et al. (2012), by implementing a novel information structure manipulation where we make one referent (the object or subject) given in the discourse before presenting the sentence. In Experiment 1, the input language had flexible word order and optional object case marking unconditioned on information structure. We found that information structure impacted word order (given objects gave rise to increased OSV sentences) and that word order impacted object marking (OSV increased object marking). However, we found no evidence of a direct impact of information structure on case marking. In Experiment 2, there was again optional case marking, but word order was fixed, and thus could not mediate the relationship between information structure and case marking. Again, we found no evidence for a direct relation between information structure and object marking. These findings, therefore, fail to confirm the predictions of a predictability‐based marking account of the emergence of DOM, at least with respect to atypical information structure.

Importantly, however, the pattern we found is in line with documented cases of DOM emergence in natural languages (Downing, 2018; Iemmolo, 2010, 2013). In these cases, a tendency to put given information earlier in the sentence than its canonical position leads to the use of noncanonical word order, in which the fronted object receives additional marking. Although we did not set out to test this pathway of change, we replicated it in the lab. In doing so, our results point to a more nuanced impact of information structure on DOM than the direct link proposed in current formulations of a predictability‐based marking account for DOM. Various linguists have advocated for the role of atypical information structure on the emergence of DOM (Cristofaro, 2013, 2019; Dalrymple & Nikolaeva, 2011; Iemmolo, 2010, 2013; König, 2008); a subset of these have argued that word order has an important role in this process (Escandell‐Vidal, 2009; Iemmolo, 2010, 2013). While the two accounts are typically lumped together (as highlighting the role of information structure), our results support the latter version: rather than being marked directly because of their atypicality (Haspelmath, 2019, 2021b, 2021a; Levshina, 2021), information structure impacts word order (i.e., a dislocation of the object), leading to marking of the object now appearing in a noncanonical position (Iemmolo, 2010, 2013).

Interestingly, in some of the languages where DOM originates from atypical information structure, there is an additional stage in this pathway of change. After being restricted to given dislocated objects, the marking expands to other types of objects—for example, animate objects. This further step is argued to emerge due to the strong association between givenness and properties like animacy and definiteness (see Fig. 1, Cristofaro, 2013; Givón, 1976; Iemmolo, 2010, 2013). Over time, the marking is reanalyzed as being associated with animate or definite objects (a fixed property) rather than given objects (a context‐dependent property, Cristofaro, 2013, 2019; Iemmolo, 2010). In ongoing work, we are testing whether this final step, a generalization from marking of given objects (in a noncanonical position) to *semantically* conditioned DOM, can also emerge in the lab.

As discussed above, the indirect path to DOM supported by our results can be seen as being driven by two tendencies: a tendency to place given information before new, and a tendency to mark objects in a noncanonical sequential position. Both tendencies impact language processing and have been argued to influence language structure in other domains. The given‐before‐new preference is well documented both in language use (Arnold, 2016; Arnold et al., 2000) and across language systems (Birner & Ward, 2009), and has been invoked to explain why the majority of world languages have subject‐initial word orders. This is because subjects are typically given (Fig. 1), and therefore, if speakers tend to place given information first, subject‐initial word order is expected to be favored (Fenk‐Oczlon, 1989, 2001). This tendency has been argued to be driven by the speaker: retrieval (and thus production) of given information is easier because it is readily accessible in memory. By producing given information first, speakers afford themselves extra time to retrieve (and produce) less accessible new information (Arnold, 2016; Arnold et al., 2000). While our experiments did not involve communication or contrast listener versus speaker effort, our results are nevertheless compatible with this view and indeed provide the first evidence for a given‐before‐new tendency in artificial language learning.

The second tendency we observed—a tendency to increase marking for objects appearing in noncanonical positions—has been found in previous artificial language learning studies (Fedzechkina & Jaeger, 2020; Fedzechkina et al., 2012, 2017; Smith & Culbertson, 2020). In particular, we found that participants used object marking more for OSV sentences than for SOV sentences. Here, we have referred to OSV as a noncanonical sequential position; however, this deserves more consideration. Why do participants tend to increase marking for OSV sentences? One possibility is that this reflects a general cognitive preference for the first noun in the sentence to be interpreted as the subject (Bickel, Witzlack‐Makarevich, Choudhary, Schlesewsky, & Bornkessel‐Schlesewsky, 2015; Bornkessel‐Schlesewsky, Choudhary, Witzlack‐Makarevich, & Bickel, 2008; Schouwstra & De Swart, 2014). Indeed, OSV is very rare across the world's languages, suggesting that speakers might have a cognitive bias against it. A different explanation for the increased marking in OSV sentences is that this effect is driven by participants’ experience with their own native language. Participants in the current study, as in previous experiments reporting this pattern (Fedzechkina & Jaeger, 2020; Fedzechkina et al., 2012, 2017; Smith & Culbertson, 2020), were native speakers of English–an SVO language. An object‐initial sentence differs from their language experience, and therefore, speakers might prefer to increase marking for this unexpected order. The current study cannot differentiate between these two explanations. Notably, however, both of them are congruent with the general framing of predictability‐based marking. Deviations from expected associations—whether the associations derive from general cognitive biases or from L1 experience—tend to be coded by longer grammatical forms (Haspelmath, 2008, 2021a). In this sense, although we did not find a direct impact of information structure on object marking, the predictability‐based marking account is supported by the finding that atypical word order receives more marking.

Finally, to return to the initial debate posed in the introduction, our results indicate that the emergence of DOM is impacted not only by ambiguity avoidance but also by context‐sensitive, discourse‐pragmatic factors. These different pressures could all be at play (Iemmolo, 2013; Seržant, 2019), alongside other noncognitive factors, such as language contact, and genetic and areal relationships among languages (Bickel et al., 2014). Indeed, these two pressures may work together, impacting distinct aspects of the linguistic system. The need for ambiguity avoidance might be more pronounced in language *communication* than in language *learning*. This proposal has been recently put forth by Smith and Culbertson (2020), who argue that pressure for ambiguity avoidance is not relevant during learning but rather comes into play during communication. That is, ambiguity avoidance might play an important role in the formation of DOM systems, but not because of learning biases. Importantly, our study, like Fedzechkina et al. (2012), does not involve communication but only learning. Therefore, the effects we see may be driven by learning or production biases, both of which shape natural language.

An alternative possibility that we have hinted at above is that these two pressures might influence different diachronic stages of DOM. In particular, there may be a difference between the forces driving the *emergence* of a linguistic system and the possible advantages this system might confer *once it is established*. Specifically, the two biases we have found in our study—a given‐before‐new bias and a bias for increasing marking of objects in noncanonical order—may drive languages to create a DOM‐type system. Once DOM is in place, speakers may utilize DOM to avoid ambiguity (Fedzechkina et al., 2012; Seržant, 2019; Smith & Culbertson, 2020). In other words, it is possible that ambiguity avoidance plays a role in maintaining or regularizing DOM systems once they exist, even if their initial emergence is due to other cognitive pressures. Future work can explicitly investigate this possibility, for example, by using an artificial language learning paradigm in which both learning and communication are required, and where systems are transmitted over simulated generations of learners (i.e., iterated learning paradigms; Kirby, Cornish, & Smith, 2008).

More broadly, the current paper demonstrates the way in which experiments can contribute to linguistic debates and complement typological and diachronic data. First, experiments can tease apart different theories (here, the ambiguity avoidance account and the predictability account). Second, they can replicate diachronic processes (here, the emergence of DOM systems via change in word order). Experimental replications of diachronic processes are essential for the study of language change: typological studies of language change often have only limited evidence, and even more importantly, they cannot address the underlying cognitive mechanisms involved in these processes. Experimental replications thereby provide corroborating evidence for the proposed language changes and couple these processes with specific cognitive mechanisms. Finally, as evidenced in the present study, experiments can also highlight previously unnoticed properties in the current linguistic accounts. By revealing the important mediating role of word order in the formation of DOM systems, the current experiments differentiate between typological accounts that were previously grouped together. Taken together, our study joins a growing body of work demonstrating the potential of using experimental methods to study the relationship between cognitive biases and recurrent typological patterns.

## Data Availability

All data reported in this paper, as well as the scripts used to generate all reported results and figures are available at: https://osf.io/d3k8h

## Notes

1. While the parallel phenomenon of *differential subject marking*, in which atypical subjects receive increased marking, is also attested across languages (Haspelmath, 2021b; Levshina, 2021; Witzlack‐Makarevich & Seržant, 2018), we limit the current investigation to the marking of objects.

2. Importantly, Smith and Culbertson (2020) found that ambiguity avoidance *does* influence object marking in explicitly communicative tasks, rather than learning‐and‐recall tasks like those used by Fedzechkina et al. (2012). We return to this in the General discussion.

3. A similar perspective offered in the linguistic literature is that DOM serves a *local* disambiguation function, in the sense that it tends to appear with the type of referents that call for ambiguity avoidance most frequently: when encountering such referent, a speaker tends to mark it *locally* regardless of whether the sentence as a whole is *globally* ambiguous (see Seržant, 2019).

4. For the rest of the paper, we group topicality and givenness together, as the current study is not designed to differentiate between them.

5. To be more precise, the claim is that new information tends to appear either in the object position or in the subject position in intransitive clauses (marked S), where there are no objects. Here, we focus on transitive sentences only, and therefore, when mentioning subjects, we refer only to subjects in transitive sentences (marked A), in which given information typically appears.

6. In theory, information structure could be involved in ambiguity avoidance just like animacy. Note, however, that this possibility is typically not raised in the literature.

7. In the artificial language learning paradigm, it is not trivial to tease apart a semantic and a syntactic representation of word order (i.e., whether the word order in which the subject [agent] occurs before the object [patient] should be described as subject‐object‐verb or agent‐patient‐verb). For the sake of consistency, we use the syntactic notation throughout, following typological practice in word order studies (Dryer, 2013; Greenberg, 1963) and experimental studies, such as Fedzechkina et al. (2012) and Smith and Culbertson (2020).

8. These drawings were based on the stimuli from Branigan, Pickering, and Cleland (2000) and were produced by Sara Rolando.

9. We ran an additional 1‐day study to make sure that our manipulation was not understood as a manipulation of definiteness, see Appendix A.

10. We first ran a pilot version of this experiment with 50% object marking, as in Experiment 1. We found that participants radically decreased object marking in their outputs, to the extent that any effect of information structure on object marking was likely to be obscured by floor effects. We next piloted a version with 75% object marking, but then participants tended to globally *overuse* object marking. Ultimately, 62.5% object marking appears to be a suitable input proportion in which participants keep variable rates of marking in their outputs. This is necessary in order to observe potential conditioning on information structure. The impact of input structure (specifically, marker frequency) on learners’ object marking use is compatible with previous literature on regularization (Ferdinand et al., 2019). Furthermore, it suggests that the tendency to reduce object marking when it does not efficiently reduce ambiguity (Fedzechkina et al., 2017) might be challenged by other cognitive biases (see Smith and Culbertson 2020 for several additional critiques); specifically, we found in our pilot data that participants trained on 75% object marking *increased* their use of those markers despite the fact that they are redundant in a fixed word order language, presumably due to the tendency to regularize.

## Appendix A

### Translation experiment

Given objects are often also definite (see Fig. 1, Cristofaro, 2013, 2019; Givón, 1976; Iemmolo, 2010; Levshina, 2021). However, since it has been claimed that atypical information structure is the driving source of DOM (Cristofaro, 2013, 2019; Dalrymple & Nikolaeva, 2011; Iemmolo, 2010, 2013; König, 2008), we wanted to ensure that our information structure manipulation is not in fact a manipulation in definiteness.^11^ To test this, we created a 1‐day experiment to investigate participants’ treatment of the characters with respect to definiteness. We used the same paradigm as Experiment 1; however, instead of a production phase, participants had a translation task: Participants were instructed to translate the sentence from Smeespeak to English (see details below). If participants treat the information structure manipulation as a definiteness manipulation, such that the preintroduced characters are treated as definite (and the “new” characters are not), we expect that (what we call) given referents will be described using definite descriptions and new ones using nondefinite descriptions.

#### Method

##### Participants

Participants were recruited in the same procedure as in Experiment 1 for only one session (1 day). Seventeen self‐reported English‐speaking participants took part in the study and were paid $6 for their participation.

##### Input language and stimuli

The stimuli and language were identical to those used in Experiment 1.

##### Manipulation of information structure

Same as Experiment 1.

##### Procedure

Same as Experiment 1, except that this was a 1‐day experiment and that the final sentence production test phase (that appeared only on days 2–4 in Experiment 1) was replaced with a translation phase (64 trials). Similar to the sentence training phase, in each translation trial, participants saw one of the characters (with an arrow on top, see 3.1.3 and Fig. 2) followed by a transitive action, which involved the previously introduced character and another character. The transitive event was accompanied by its audio and text description in Smeespeak. This time, however, participants had to translate the sentence to English by typing into a text box.

##### Scoring

Participants’ descriptions for each subject and object were coded as definite/indefinite/bare noun/proper name (the Smeespeak name as is).

#### Results

We classified participants according to their dominant translation pattern. Interestingly, while participants varied between them in terms of their translations, each participant had a very consistent interpretation pattern, regardless of our manipulation (the least dominant translation type occurred in 86% of the trials): seven participants used bare nouns for both the subject and object (e.g., cop shoots doctor), seven used definite nouns for both (e.g., the cop shoots the doctor), one used indefinite nouns for both (e.g., a cop shoots a doctor), one used proper names for both (e.g., slagum shoots tombat), and one used bare nouns for subjects and indefinite nouns for objects (cop shoots a doctor). Importantly, although participants assigned different definite statuses to the characters, they tended to treat the subject and object equally, and in any event, the definiteness‐status of the characters was not impacted by our givenness manipulation. Across all trials of all participants (64 trials×17 participants), there were only seven trials in which the given referent was treated as definite, whereas the new referent was not. For example, a case in which *dancer…boxer‐kick‐dancer* was translated as “boxer kicked the dancer.” We take the paucity of these translations as an indication that the givenness manipulation was *not* perceived as a definiteness manipulation.
